# Dr. D. R. Fly

**Published:** 1912-01

**Authors:** 


### DR. D. R. FLY,

PRESIDENT TEXAS STATE MEDICAL ASSOCIATION.

After an illness of several weeks, Dr. D. R. Fly died in St. Joseph's Sanitarium, Fort Worth, December 10th ult. His home was in Amarillo, and he had visited Marlin and other places in the State recently in hope of some relief. He was but little over 45 years of age. He was at the time of his death President of the State Medical Association and for some years had been a leader in the successful movement for State aid for the sufferers from tuberculosis and for other methods of improving sanitary conditions in Texas.

In 1899 Dr. Fly was married to Miss Lizzie Miller of Dallas, daughter of Mrs. Rachel E. Miller, and sister of Hon. Barry Miller. She survives him, as do his two brothers, Dr. A. W. Fly of Galveston, member of the State Board of Health and of the medical board of his city; W. C. Fly of Fort Worth and two half-brothers, Robert C. Fly of Amarillo and DeWitt C. Fly of California.

At the annual meeting of the State Medical Association in Amarillo, May 11, 1911, Dr. Fly was made president of the organization. Shortly afterward there appeared in the *Texas State Journal of Medicine* the following account of the man:

"Dr. D. R. Fly of Amarillo was elected the forty-third president of the State Medical Association of Texas at the Amarillo meeting, May 11, 1911.

"Dr. Fly was born near Water Valley, Yaloubusha county, Mississippi, October 15, 1865. He received his preliminary education in the public schools of his native county, where he resided until his removal to Galveston, Texas, in 1884. He began his medical career in 1883 as 'chief bottle washer' in a country drug store, continuing his apprenticeship in Galveston immediately upon his arrival at that place. In 1888 he opened a drug business for himself, selling out in 1892, and removing to the Panhandle of Texas for his health. Deciding to make the practice of medicine his life work, Dr. Fly entered the Kentucky School of Medicine in 1893, graduating with the class of 1894.

"Immediately after his graduation he moved to Fort Worth, where he entered general practice. Upon the organization of the Medical Department of Fort Worth University he became demonstrator of anatomy and chief of clinic in that institution, which position he held until his health again forced him to remove to the Panhandle in 1900. He held the position of City Physician of Fort Worth from 1895 to 1897.

Dr. Fly continued his teaching work in his new home, Amarillo, lecturing for two years on physiology and hygiene in the Amarillo College. He has been a visiting and consulting member of the medical staff of the Hospital of the Sisters of the Incarnate Word, for the location of which in Amarillo he was largely responsible. He is now local surgeon of the Rock Island and Fort Worth & Denver railroads, and consulting surgeon for the Santa Fe road.

"Dr. Fly has long been interested in organized, scientific medicine. He was one of the organizers of the old Panhandle District Medical Association, and for six years its president. He was elected councilor of the Panhandle district at the time of the reorganization at San Antonio, in 1903, which position he has held continuously since that time. He was successful in this work from the beginning, organizing his district as well as its sparsely settled condition would permit, and bringing about the affiliation of the Panhandle District Association with the State Association in 1905. In addition to his other duties, Dr. Fly served as chairman of the committee on public lectures for a year, devoting four months of his time to this work exclusively.

"It will be observed that our new president is no stranger to the work, and it is his boast that he is no stranger to the profession of the State many of whom he holds as his warm personal friends."—*Dallas News*.

